# Bonding of Metal Orthodontic Attachments to Sandblasted Porcelain and Zirconia Surfaces

**DOI:** 10.1155/2016/5762785

**Published:** 2016-09-22

**Authors:** Amitoj S. Mehta, Carla A. Evans, Grace Viana, Ana Bedran-Russo, Maria Therese S. Galang-Boquiren

**Affiliations:** ^1^Department of Orthodontics, University of Illinois at Chicago, Chicago, IL, USA; ^2^Department of Restorative Dentistry, University of Illinois at Chicago, Chicago, IL, USA

## Abstract

This study evaluates tensile bond strength (TBS) of metal orthodontic attachments to sandblasted feldspathic porcelain and zirconia with various bonding protocols. Thirty-six (36) feldspathic and 36 zirconia disc samples were prepared, glazed, embedded in acrylic blocks and sandblasted, and divided into three groups according to one or more of the following treatments: hydrofluoric acid 4% (HF), Porcelain Conditioner silane primer, Reliance Assure® primer, Reliance Assure plus® primer, and Z Prime™ plus zirconia primer. A round traction hook was bonded to each sample. Static tensile bond strength tests were performed in a universal testing machine and adhesive remnant index (ARI) scoring was done using a digital camera. One-way ANOVA and Pearson chi-square tests were used to analyze TBS (MPa) and ARI scores. No statistically significant mean differences were found in TBS among the different bonding protocols for feldspathic and zirconia, *p* values = 0.369 and 0.944, respectively. No statistically significant distribution of ARI scores was found among the levels of feldspathic, *p* value = 0.569. However, statistically significant distribution of ARI scores was found among the levels of zirconia, *p* value = 0.026. The study concluded that silanization following sandblasting resulted in tensile bond strengths comparable to other bonding protocols for feldspathic and zirconia surface.

## 1. Introduction

Advancements in cosmetic dentistry and orthodontic treatment needs in the United States have led to increasing number of adult patients seeking orthodontic care [1]. Orthodontists must often bond attachments to various dental restorations including those fabricated with porcelain. Feldspathic, leucite, and lithium disilicate are the silica based porcelains while zirconia is a nonsilica based ceramic. Feldspathic porcelain is the most esthetic porcelain due to its high translucency. Zirconia is one of the most popular types of all ceramic restorations today [2]. For the different types of porcelains, literature suggests a wide range of surface treatments that include sandblasting, hydrofluoric acid treatment, silane application, or a combination of them. For feldspathic and lithium disilicate-based porcelain the combination of sandblasting and silane has shown highest shear bond strength values in comparison to sandblasted only samples [3, 4]. For surface treatment of zirconia, application of primer containing 10-methacryloyloxydecyl dihydrogen phosphate (10-MDP) after air abrasion has been recommended [5–7]. The use of ceramic primer along with self-adhesive resin composite cement has shown a positive effect on shear bond strength to zirconia and is recommended during crown cementation [8]. Manufacturers have introduced different primers for feldspathic and zirconia crowns. However, when orthodontic providers wish to bond an attachment to an esthetic crown, it is difficult to differentiate clinically a feldspathic porcelain crown from a zirconia crown. Recently, products such as one-step universal primer for silica based and nonsilica based porcelains have been introduced in the market. One example is Reliance Assure plus (RA plus) (Reliance Orthodontic Products, Itasca, IL), with a chemical composition of BisGMA, bisphenol A-glycidyl methacrylate, and ethanol. It is imperative for clinicians to know the differences in bond strengths obtained with various available bonding protocols.

Orthodontic bonding primarily involves three steps: etching, primer application, and bonding [9]. To minimize the number of clinical steps, self-etching primers and one-step universal primers were introduced. These primers are composed of unfilled resin and play an indispensable role in the bonding process. With the introduction of self-etch adhesives the number of clinical steps was reduced. Self-etch adhesives do not require rinsing the enamel surface with water. The treated smear layer and demineralized products are incorporated into the resin [10]. Zachrisson et al., Kocadereli et al., and Abu Alhaija et al. all suggested sandblasting to increase the bond strength to porcelain surface [11–13]. However, it has been reported that mechanically roughening the surface of porcelain also entails a higher incidence of porcelain surface fracture [14]. Hydrofluoric acid (HF) has been advocated as a chemical treatment of the porcelain surface to increase the bond strength [11, 12]. Hydrofluoric acid attacks the glass phase of ceramics, inducing microporosities leading to micromechanical bonding with composite resin. However, Hayakawa et al. reported the corrosive nature of hydrofluoric acid causing damage to oral tissues [15]. Bach et al. conducted a systematic review on orthodontic bonding to porcelain; they discussed that application of silane increases the bond strength of brackets to porcelain surfaces [16].

Bonding orthodontic attachments to zirconia surfaces differs in bonding protocol from that of the feldspathic crown surfaces. In contrast to feldspathic porcelain, zirconia does not contain a glass phase and etching zirconia surface with hydrofluoric acid does not enhance bond strength [17, 18]. A systematic review concluded that increased adhesion to zirconia is expected after physicochemical conditioning that involves combination of air abrasion and adhesive promoters such as primers or silanes [19]. Chen et al. concluded that the bond strength of resin cement to zirconia is increased by silanization of the zirconia surface [20]. Another study concluded that incorporation of BisGMA into silane containing zirconia primers does not affect their efficacy while it has shown to affect the bonding ability of silane containing primers [21].

This study aims to investigate different bonding procedures for metal orthodontic attachments to feldspathic porcelain and zirconia surfaces.

## 2. Materials and Methods

### 2.1. Sample Size and Preparation

A total of 36 zirconia specimens, 97% zirconium dioxide stabilized with a 3% Yttria-Lava Frame (Sagemax Bioceramics, WA), was obtained. The blocks were sectioned with diamond blade (Allied High Tech Productions Inc., Compton, CA) to obtain 2.5 mm thick and 8.5 mm in diameter disc specimens. The samples were polished using 600-grit silicon carbide paper and subsequently water-cooled. The blocks were then ultrasonically cleansed and glazed in a Dekema oven (Freilassing, Germany) at 1500°C for one minute.

A total of 36 glazed feldspathic porcelain specimens measuring 2 mm thick and 8.5 mm in diameter were prepared. Feldspathic porcelain powder (GC America, Alsip, Illinois) was stacked onto degassed metal disks and fired in a Dekema oven (Freilassing, Germany) at a temperature of 895°C. The sintered specimens were ultrasonically cleaned and glazed.

### 2.2. Surface Treatment

All the samples were embedded in acrylic (Orthodontic Resin, Item # 040–013, Great Lakes Orthodontics Ltd., Tonawanda, NY), placed in mounting blocks, and sandblasted with 50 *μ*m aluminium oxide particles at 30 psi for 4 seconds in a Basic Meter Sandblaster (Renfert Corp, Hilzingen, Germany). The ideal protocol for air abrasion is sandblasting with aluminium oxide particles for 4 sec at 2.5-bar pressure (36.5 psi) [16].

Each material was divided into three groups (Tables 1 and 2) according to one or more of the following surface treatments: hydrofluoric acid 4% (HF), Porcelain Conditioner (silane) (Reliance Orthodontics, Itasca, IL) (trimethoxysilyl-propyl-2-methyl-2-propenoic acid and acetone), Reliance Assure (RA) (Reliance Orthodontics) primer (biphenyl dimethacrylate, hydroxymethyl acrylate), Reliance Assure plus (RA plus) (Reliance Orthodontics) primer (bisphenol A-glycidyl methacrylate and ethanol), and Z Prime plus (Bisco, Inc., Schaumburg, IL) zirconia primer (biphenyl dimethacrylate, methacryloyloxydecyl dihydrogen phosphate, and ethanol).

### 2.3. Bonding Adhesives

Four different kinds of bonding adhesives (Table 3) were used in the study. All four have different chemical properties and application techniques.

1. Reliance Assure (RA) primer: the bonding protocol following the manufacturer instructions is to sandblast the porcelain surface with aluminium oxide particles for 4 sec, followed by surface treatment with 4% hydrofluoric acid for 60 sec, rinsing with water and light air drying, applying a single coat of silane, and light air drying for 3–5 sec. Lastly a single coat of the RA primer is applied before using the adhesive for bonding.
2. Reliance Assure plus (RA plus) primer: manufacturers suggest sandblasting with aluminium oxide particles for 4 sec, followed by application of a single coat of the RA plus primer and light air drying for 3–5 sec before bonding to porcelain and zirconia surfaces. Per the manufacturer, this bonding protocol has reduced number of clinical steps involved in surface preparation for bonding to porcelain and zirconia surfaces.
3. Z Prime plus (Bisco) primer: the bonding protocol following the manufacturer instructions includes sandblasting with aluminium oxide particles for 4 sec, followed by a single coat application of Z Prime plus primer and light air drying for 3–5 sec before bonding to zirconia.
4. Porcelain Conditioner silane primer: it was suggested by the manufacturer to be used as one of the preliminary bonding protocol steps to condition feldspathic porcelain. In this study it is a control to the other bonding protocols.

### 2.4. Bonding Attachments

Following the surface intervention, a round traction hook with a laminated mesh bonding pad, Product # 224–011 from TP Orthodontics (La Porte, Indiana), was bonded using Pad Lock™ light cure composite (Reliance Orthodontics, Itasca, IL). The bonding surface of the traction hook measured 9.95 mm^2^. For consistency, all the attachments were bonded to the samples by the same clinician. Each sample was light cured for 20 seconds from the center of the sample to its rim with Ortholux light at 900–1,100 mW/cm^2^ (3 M Unitek, Monrovia, CA). Light intensity was measured using a curing light intensity meter, Model #8000 (EFOS Inc., Mississauga, Ontario, Canada) for standardization.

### 2.5. Tensile Bond Strength Testing

Feldspathic porcelain and zirconia samples were stabilized in the acrylic blocks and ensured for parallelism using stainless steel ruler. Using a 12 V cordless drill (Black and Decker, Towson, Maryland), holes measuring 5/32 inches were drilled into the acrylic units. All the samples were labeled with material group (feldspathic or zirconia) and bonding agent group (RA, RA plus, Z Prime plus, and Porcelain Conditioner). Samples were randomized into three batches constituting four samples from each bonding protocol. Each batch was prepared according to the respective bonding protocol and tested for tensile bond strength measurement immediately. This helped minimize bias with environmental conditions and clinician efficiency.

A round 0.012-inch stainless steel wire was used to loop around the traction hook. A perpendicular tensile force was ensured by attaching this wire to the upper unit of the Instron testing machine Model 1125 (Instron Corp., Canton, MA). The acrylic units were secured via a rod to the lower unit of the Instron machine. The Instron machine was directed to provide tensile stress to the bracket unit until bond failure occurred at a cross head speed of 1.0 mm/minute.

Machine calibration was performed each time a batch was tested and the load balance was set at zero to ensure uniformity. The size load cell for the testing was set to be at 500 kg. Once the sample to be tested was engaged with the stainless wire looping around the traction hook and attached to the upper unit the test was run. The data collection was done using TestWorks® software (MTS Corp., Eden Prairie, MN). The bond strength measured was calculated using the formula *R* = *F*/*A*, where “*R*” is the strength (MPa), “*F*” is the load required for rupture of the specimen (*N*), and “*A*” is the interface area of the specimen (mm square). A digital caliper was used to measure the interface surface area of the specimen. The surface area of the bondable surface of the traction hook measured to be 9.95 mm^2^.

### 2.6. Adhesive Remnant Index Scoring

To determine the adhesive remnant index (Artun and Bergland, 1984), a digital camera Model GT800 (Belmont, MA) was used to examine the debonded surface and the bracket mesh. The scores are as follows:

 Score 0: no adhesive left on the porcelain or zirconia surface.
 Score 1: less than half of the adhesive left on the porcelain or zirconia surface.
 Score 2: more than half of the adhesive left on the porcelain or zirconia surface.
 Score 3: almost all adhesive left on the porcelain or zirconia surface, with distinct impression on the attachment mesh.

The digital camera Model GT800 used in this study has a magnification up to 700x (digitally). An optical magnification of 230x was deemed sufficient for this study's purpose. To minimize bias all images were randomly selected and scored for ARI indices by three calibrated examiners. In case the individual scores differed amongst the examiners the majority score was chosen to be the final score.

### 2.7. Statistical Analysis

The Shapiro-Wilk test was used to check the distribution of raw data and descriptive statistics were calculated. To determine the differences in mean tensile bond strengths amongst the different bonding agents for feldspathic and zirconia samples, a one-way ANOVA was used. ARI scores were reported for each group. A cross-tabulation followed by Pearson chi-square test was done for ARI scores among the bonding agents for feldspathic and zirconia. Statistical significance was set at 0.05. Data analysis was performed using IBM SPSS Statistics for Windows (version 22.0 IBM Corp., Armonk, NY).

## 3. Results and Discussion

All study data showed normal distribution. Descriptive statistics were presented for feldspathic samples in Table 4 and zirconia samples in Table 5. The results of one-way ANOVA indicated no statistical significant mean difference on the variable tensile bond strength (MPa) amongst bonding agents on feldspathic and zirconia samples used in the study: feldspathic, *F*(2,33) = 1.029, *p* value = 0.369, and zirconia, *F*(2,33) = 0.058, *p* value = 0.944.

The literature on minimum bond strengths is not consistent. Some authors reported the minimum shear bond strength for orthodontic bonding purpose to be 13 to 21.3 MPa [22], while some authors reported the minimum tensile bond strength to be between 6 and 8 MPa [23, 24]. Bond strengths as low as 3–5 MPa have been reported as well [25]. The reason for the differences between the literature and this study may be explained on the basis of (1) the method of bond strength measurement, (2) the time duration from bonding the brackets to the time the actual testing was performed, and (3) the protocol for curing the bracket adhesive. The literature search suggests shear bond strength testing to produce higher values than tensile bond strength tests [26]. In this study 12 samples were used for each intervention group. A minimum of 10 specimens is recommended to perform the shear bond strength testing [27]. Previous studies had specimens thermocycled to induce mechanical fatigue. However, from a clinical standpoint, immediate bond strength is important since arch wires exerting a force are engaged into the brackets within minutes of the bonding procedure.

Feldspathic porcelain (silica based) undergoes surface roughness after sandblasting and/or exposure to hydrofluoric acid, exposing silica oxides for chemically bonding with silane coupling agent and resin. The silanol (Si-OH) group of the primer and the OH group of the ceramic combine to liberate a water molecule and in the process form a stable siloxane (Si-O-Si) bond [28, 29]. It has been reported that sandblasting increases the surface roughness of zirconia [30]. The importance of sandblasting in increasing the bond strength between the adhesive resin and the sample is supported by the study conducted by Ourahmoune et al. [31]. They observed that sandblasting increases surface roughness and the wettability behavior for all materials is significantly influenced by their surface morphology. The contact angle increased with average particle size of the aluminium oxide particles, increasing the mechanical interlocking and hence improving the bond strength. It can be interpreted that sandblasting leads to an increased surface roughness and an increased contact angle of wettability, following which any of the bonding protocols included in this study can provide tensile bond strength values that are not statistically different.

While debonding brackets from enamel, it is important to prevent enamel damage but at the same time have minimal adhesive left on the tooth surface. Likewise, for ceramics, the aim is a debond site that has minimal cohesive damage to porcelain or zirconia but at the same time has minimal residual composite left to remove.

The ARI scores for various bonding agents on feldspathic group and in zirconia group are shown in Tables 6 and 7. For feldspathic group the Pearson chi-square results indicated that Reliance Assure plus, Reliance Assure, and Porcelain Conditioner are not significantly different on whether they were scored as 1, 2, or 3, *χ* ^2^ = 2.934, df = 4, *N* = 36, *p* value = 0.569. For zirconia group the Pearson chi-square results indicate that Reliance Assure plus, Z Prime, and Porcelain Conditioner are significantly different on whether they were scored as 1, 2, or 3, *χ* ^2^ = 11.059, df = 4, *N* = 36, *p* value = 0.026.

This type of failure mode signifies that the physiochemical bond that was formed between the various bonding agents and the sandblasted feldspathic and zirconia samples in this study was higher than the micromechanical retention between the adhesive and the traction hook base. It can be interpreted that the traction hook base was not retentive enough for the adhesive and before the bond strength limit could be reached for any of the samples the traction hook debonded. This may also be purely intentional on the part of the manufacturer because bond strength that is significantly high may pose a risk to harming tooth enamel; thus, by design, the bracket is supposed to debond at the bracket-adhesive interface. Therefore, with the current study design it cannot be determined if there was no difference in tensile bond strength of orthodontic attachments to sandblasted feldspathic and zirconia samples with the included bonding protocols.

The limitation of this study is no inclusion of a material group that was not sandblasted. It is recommended that future studies include a control group where a nonsandblasted surface is tested for application of various bonding protocols.

## 4. Conclusion

From the results obtained in this study it can be concluded that bonding metal orthodontic attachments to sandblasted surfaces of feldspathic porcelain and zirconia with Porcelain Conditioner, Reliance Assure (RA), Reliance Assure plus (RA plus), and Z Prime plus resulted in tensile bond strengths that were higher than the micromechanical retention between the adhesive and the orthodontic attachment hook base.

## Figures and Tables

**Table 1:** Surface treatment for the feldspathic porcelain group.

Bonding protocols	Surface treatment
Group 1	Sandblasting + HF + Porcelain Conditioner (silane) + RA (primer)
Group 2	Sandblasting + Porcelain Conditioner (silane) + RA plus (primer)
Control group	Sandblasting + Porcelain Conditioner (silane)

**Table 2:** Surface treatment for the zirconia group.

Bonding protocols	Surface treatment
Group 1	Sandblasting + Z Prime plus (primer)
Group 2	Sandblasting + RA plus (primer)
Control group	Sandblasting + Porcelain Conditioner (silane)

**Table 3:** Composition of bonding adhesives.

Bonding adhesive	Chemical composition
Reliance Assure (RA) primer	2-Hydroxyethyl methacrylate (10–30%) and acetone (50–75%)
Reliance Assure plus (RA plus) primer	Bisphenol A-glycidyl methacrylate (10–30%) and ethanol (50–75%)
Z Prime plus (Bisco) zirconia primer	Biphenyl dimethacrylate, methacryloyloxydecyl dihydrogen phosphate (1–5%), and ethanol (70–90%)
Porcelain Conditioner silane primer	Trimethoxysilyl-propyl-2-methyl-2-propenoic acid and acetone

**Table 4:** Descriptive statistics of tensile bond strengths for bonding agents in feldspathic group.

Bonding agents	*N*	Mean (MPa)	Std. deviation	Std. error	95% confidence interval for mean	
					Lower bound	Upper bound
Reliance Assure plus	12	4.657	0.6020	0.1738	4.275	5.040
Reliance Assure	12	4.724	0.7466	0.2155	4.250	5.199
Porcelain Conditioner	12	4.339	0.7494	0.2163	3.863	4.815

Total	36	4.574	0.7033	0.1172	4.336	4.812

**Table 5:** Descriptive statistics of tensile bond strengths for bonding agents in zirconia group.

Bonding agents	*N*	Mean (MPa)	Std. deviation	Std. error	95% confidence interval for mean	
					Lower bound	Upper bound
Reliance Assure plus	12	5.323	0.5249	0.1515	4.990	5.657
Porcelain Conditioner	12	5.290	0.7287	0.2104	4.827	5.753
Z Prime plus	12	5.245	0.3900	0.1126	4.997	5.493

Total	36	5.286	0.5499	0.0916	5.100	5.472

**Table 6:** Adhesive remnant index scores cross-tabulations for bonding agents in feldspathic group.

Bonding agents	ARI scores			Total
	Score 1	Score 2	Score 3	
Reliance Assure plus	1	3	8	12
Reliance Assure	1	6	5	12
Porcelain Conditioner	0	4	8	12

Total	2	13	21	36

**Table 7:** Adhesive remnant index scores cross-tabulations for bonding agents in zirconia group.

Bonding agents	ARI scores			Total
	Score 1	Score 2	Score 3	
Reliance Assure plus	0	4	8	12
Porcelain Conditioner	1	3	8	12
Z Prime plus	0	10	2	12

Total	1	17	18	36
